# [Expression of Concern] MicroRNA-29c inhibits proliferation and promotes apoptosis in non-small cell lung cancer cells by targeting VEGFA

**DOI:** 10.3892/mmr.2025.13785

**Published:** 2025-12-22

**Authors:** Shijuan Zhan, Chunfeng Wang, Fangqing Yin

Mol Med Rep 17: 6705–6710, 2018; DOI: 10.3892/mmr.2018.8678

Following the publication of the above paper, it was drawn to the Editor's attention by a concerned reader that western blot data appeared to have been assembled incorrectly in Fig. 4A on p. 6709. In this case, there was an apparent inversion of the p-PI3K bands, and inclusion of one of these bands as a unique band (upside down) for the Control experiment in the p-Akt row of data, purportedly showing the results of a different set of experiments. The authors were contacted by the Editorial Office to offer an explanation for this apparent anomaly in the presentation of the data in this paper; however, up to this time, no response from them has been forthcoming. Owing to the fact that the Editorial Office has been made aware of potential issues surrounding the scientific integrity of this paper, we are issuing an Expression of Concern to notify readers of this potential problem while the Editorial Office continues to investigate this matter further.

